# Hamstrings and quadriceps muscle size and strength in female and male elite competitive alpine skiers

**DOI:** 10.3389/fphys.2024.1444300

**Published:** 2025-01-09

**Authors:** Daniel P. Fitze, Martino V. Franchi, Clarissa Müller Brusco, Nadine Engeler, Walter O. Frey, Jörg Spörri

**Affiliations:** ^1^ Department of Orthopaedics, Sports Medical Research Group, Balgrist University Hospital, University of Zurich, Zurich, Switzerland; ^2^ Department of Orthopaedics, University Centre for Prevention and Sports Medicine, Balgrist University Hospital, University of Zurich, Zurich, Switzerland; ^3^ Human Neuromuscular Physiology Lab, Department of Biomedical Sciences, University of Padua, Padua, Italy

**Keywords:** muscle size, muscle strength, athletes, alpine ski racing, injury prevention

## Abstract

Competitive alpine skiing requires a high level of physical fitness to perform sport-specific manoeuvres and to minimise the risk of injury. The aim of this study was to establish reference values for the maximal anatomical cross-sectional area (ACSA_max_) of the individual hamstrings (HAM) and quadriceps (QUAD) muscles as well as for the maximal voluntary torque (MVT) during knee flexion (KF) and knee extension (KE) of female and male elite competitive alpine skiers. Ultrasound and dynamometer data were obtained from a largely overlapping but not identical dataset. The ultrasound data were collected from 33 elite alpine skiers (20 women and 13 men), and the dynamometer data were collected from 35 elite alpine skiers (20 women and 15 men). Compared with female skiers, male skiers presented a significantly greater ACSA_max_ in the biceps femoris short head (BFsh), biceps femoris long head (BFlh), and semitendinosus (ST) muscles, as well as in the entire HAM muscle group. The ACSA_max_ of the semimembranosus (SM) did not differ significantly between the two sexes. Compared with female skiers, male skiers presented significantly greater ACSA_max_ values in the vastus lateralis (VL), rectus femoris (RF), vastus medialis (VM) and entire QUAD muscle groups. At VI, there was no significant difference in the ACSA_max_ between the two sexes. Compared with male skiers, female skiers had a significantly greater proportional SM ACSA_max_. In terms of MVT, male skiers presented greater absolute and relative values than females did. There were no differences in the MVT/ACSA_max_ between the sexes. Neither the HAM/QUAD ACSA_max_ ratio nor the KF/KE MVT ratio differed between the sexes. The present study provides normative values for the muscle size and strength of the HAM and QUAD muscles of elite competitive alpine skiers. These values can be used as benchmarks for youth alpine skiers striving for the elite level. An interesting finding of the present study was that female skiers had a greater proportional ACSA_max_ of the SM, as this may be relevant in anterior cruciate ligament injury prevention given the function of tibia internal rotation.

## 1 Introduction

Competitive alpine skiing requires a high level of physical fitness to perform sport-specific manoeuvres and to minimise the risk of injury. The combination of high speeds (Bere and Bahr, 2014) and high ground reaction forces (Spörri et al., 2022) places high demands on the lower extremities (Spörri et al., 2017; Jordan et al., 2017). The knee joint is exposed to extreme kinematics (Zorko et al., 2015), and the interaction with the snow causes vibration loads and perturbations, which must be absorbed by the knee and its stabilising muscles (Supej et al., 2018). Injuries to the lower extremities are therefore common and the knee joint is one of the most frequently injured body parts in youth (Schoeb et al., 2020) and elite (Fröhlich et al., 2021) competitive alpine skiers. Tears of the anterior cruciate ligament (ACL) are the most commonly diagnosed type of traumatic knee injury (Barth et al., 2020). The influence of sex on the risk of ACL injury has not been fully elucidated. While earlier epidemiological studies of competitive alpine skiers at the World Cup level revealed no differences in ACL injury rates between sexes (Florenes et al., 2009; Bere et al., 2014), more recent studies revealed that the risk of injury is increased in female adolescents (Raschner et al., 2012) and elite (Barth et al., 2020) alpine skiers. Regardless of the potential influence of sex, ACL injuries lead to substantial time loss in training and competition, surgery and/or rehabilitation for several months. In the worst case, such an injury history can lead to a career ending.

From a functional point of view, the ACL antagonises anteriorly directed shear forces and internal rotation forces of the tibia relative to the femur (Duthon et al., 2006). Mechanisms in which the tibia is translated forward relative to the femur and where the knee is forced into dynamic knee valgus and the tibia rotates internally are typical for rupture of the ACL in competitive alpine skiing (Spörri et al., 2017; Jordan et al., 2017). Turning and jump landings are therefore crucial sport-specific manoeuvres. Recent studies (Heinrich et al., 2023a; Heinrich et al., 2023b) suggest that the neuromuscular control of the hamstrings (HAM) can influence the load on the ACL in such situations. For example, the HAM are among the primarily activated muscles of the outer leg during turning, where the ACL load increases significantly (Heinrich et al., 2023a). Furthermore, it has been shown that the load on the ACL during jump landing can be minimised by earlier and greater activation of the HAM in addition to other neuromuscular control strategies (Heinrich et al., 2023b). In contrast to the role of the HAM as possible ACL synergists (MacWilliams et al., 1999), the quadriceps (QUAD), especially in the range of motion close to full knee extension, can load the ACL (DeMorat et al., 2004). Therefore, the force capacity of the HAM muscles in relation to the QUAD muscles is an important modifiable variable for the prevention of ACL injuries in alpine skiing (Spörri et al., 2017; Jordan et al., 2017).

While there have been numerous studies on maximal voluntary torque (MVT) during knee flexion (KF) and knee extension (KE) in elite competitive alpine skiers (Spörri et al., 2017; Jordan et al., 2017), few data are currently available on the individual HAM and QUAD muscle sizes, despite their potential effects on the ACL load. Therefore, the aim of the present study was to investigate the size (i.e., maximal anatomical cross-sectional area (ACSA_max_)) and strength (i.e., KF and KE MVT) profiles of the individual hamstrings and quadriceps muscles in female and male elite competitive alpine skiers.

## 2 Methods

### 2.1 Participants and ethics

To participate in this study, skiers had to be members of a national ski squad, not have any “unhealed” injuries that still restrict their sports participation, or have undergone surgery in the previous 12 months. The ultrasound and dynamometer data originated from a largely overlapping but not identical dataset. This occurred because the ultrasound and dynamometry measurements were performed on two different occasions within 3 months during the preseason period. Among the elite alpine skiers included, 28 (18 females and 10 males) participated in both measurements, but some participated on only one of the two testing days. As a result, ultrasound data were collected from 33 elite alpine skiers (20 females and 13 males), and dynamometry data were collected from 35 elite alpine skiers (20 females and 15 males). The anthropometric measurements of all study participants are shown in Table 1. Participation was voluntary, and all participants provided written informed consent. The study was conducted in accordance with the Declaration of Helsinki, and the underlying protocol was approved by the local ethics committee of the Canton of Zurich (KEK-ZH-NR: 2017-01395).

**TABLE 1:** Anthropometric measurements of the study participants.

	Ultrasound data (n = 33)		Dynamometer data (n = 35)	
	Female (n = 20)	Male (n = 13)	Female (n = 20)	Male (n = 15)
Age (y)	21.5 ± 2.6	23.0 ± 2.5	21.2 ± 2.4	22.6 ± 2.6
Body height (cm)	167.0 ± 5.3	177.5 ± 6.2	168.2 ± 6.8	177.6 ± 6.4
Body mass (kg)	67.1 ± 5.6	82.7 ± 8.1	67.1 ± 7.9	82.0 ± 8.7
BMI (kg·m^−2^)	24.0 ± 1.7	26.2 ± 1.6	23.7 ± 1.5	26.0 ± 1.8

The data are expressed as the mean ± standard deviation. BMI, body mass index.

### 2.2 Ultrasound measurements

All ultrasound measurements were performed by an experienced operator (MVF) using the Aixplorer Ultimate system (SuperSonic Imagine, Aix-en-Provence, France) and the linear transducer SuperLinear SL10-2 (SuperSonic Imagine, Aix-en-Provence, France). For the HAM muscles, the participants were instructed to lie prone with their knees extended and their feet placed on the edge of the bed so that the ankles could be kept in a neutral position. By palpation, the anatomical reference points (i.e., the greater trochanter and lateral femoral condyle) were identified, and the resulting distance was defined as the femur length. The skin was marked at 30%, 40%, 50%, and 60% of the femur length. Using the panoramic mode, transverse images were acquired at all marks. The transducer was moved from the lateral to the medial border of the HAM muscles (i.e., the biceps femoris short head (BFsh), biceps femoris long head (BFlh), semitendinosus (ST) and semimembranosus (SM)) in a slow, controlled and low-pressure manner. For the QUAD muscles (i.e., the vastus lateralis (VL), rectus femoris (RF), vastus medialis (VM) and vastus intermedius (VI)), the participants were instructed to lie supine. The same marks (i.e. 30%–60% of the femur length) were used for the transverse panoramic scans. Because the VM requires a greater image depth, particularly at the 30% and 40% measurement sites, it was scanned individually. For all scans, transmission gel was used for better acoustic contact and to minimize the pressure of the transducer on the skin.

**FIGURE 1:**
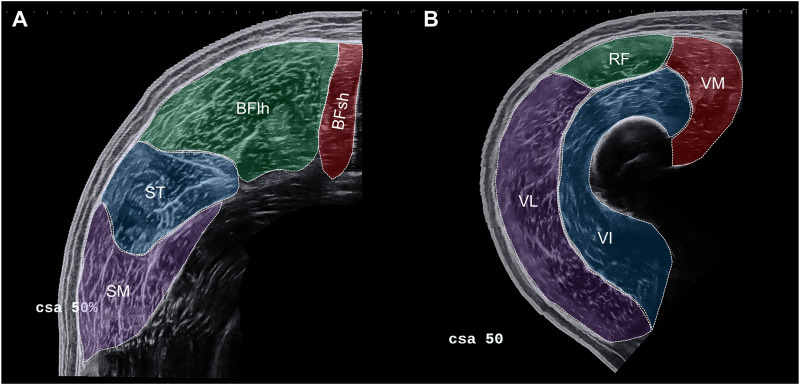
Representative transversal panoramic mode ultrasound images at 50% of the femur length with marked individual hamstrings **(A)** and quadriceps muscles **(B)**. BFsh: biceps femoris short head, BFlh: biceps femoris long head, ST: semitendinosus, SM: semimembranosus, VL: vastus lateralis, RF: rectus femoris, VM: vastus medialis, VI: vastus intermedius.

Using the freely available image processing software ImageJ (National Institutes of Health, Bethesda, MD), all image analyses were performed by an experienced rater (DPF). At each site relative to femur length (i.e., 30%, 40%, 50%, and 60%), ACSAs were analysed in randomised order by tracing the contours of the hamstring (i.e., BFlh, BFsh, ST and SM) and quadriceps (i.e., VL, RF, VM and VI) muscles. Figure 1 illustrates representative transversal panoramic mode ultrasound images at 50% of the femur length with marked individual hamstrings (A) and quadriceps muscles (B). The largest ACSA from each of the four measurement sites was defined as ACSA_max_ and considered for statistical analysis. Like in the study by Behan et al. (Behan et al., 2018), the relative proportion (i.e., proportional ACSA_max_) of the individual HAM and QUAD muscles to the corresponding muscle group was also calculated as follows and considered for statistical analysis (exemplary for the VL):

$$VL\;proportional\;{ACSA}_{\max}=\frac{{VL\;ACSA}_{\max}}{{QUAD\;ACSA}_{\max}}\times 100$$

In addition, the HAM/QUAD ACSA_max_ ratio was calculated for statistical analysis.

### 2.3 Isokinetic dynamometer measurements

The maximal voluntary torque (MVT) produced during knee flexion (KF) and knee extension (KE) was measured with the isokinetic dynamometer CON-TREX®MJ (CMV AG, Dübendorf, Switzerland). The backrest of the dynamometer was set to an inclination of 85°, the distance between the knee joint and the edge of the seat was two finger widths, and the axis of rotation was lateral to the lateral epicondyle. The starting position of the leg was at 0° of knee extension, and the range of motion was up to 100° of knee extension. All measurements were performed on the right leg. The warm-up included cycling on an ergometer, maximal jumps and parts of an individual warm-up program. In addition, the participants completed a 30-s specific warm-up program on the dynamometer with an angular velocity of 60°·s-1 at 50% of their maximal effort. The measurements comprised a total of four trials per KF and KE at an angular velocity of 60°·s-1. Each trial comprised two repetitions. During the first repetition, the subjects were instructed to perform approximately 80% of their MVT and during the second repetition, the effort was maximal. The rest interval between trials was 30 s. The participants were verbally encouraged during each trial. For the statistical analysis, the highest measured MVT from all trials was considered for the KF and KE. In addition to the absolute MVT, the MVT relative to body mass (i.e., MVT/BM) and the MVT per ACSA_max_ (i.e., MVT/ACSA_max_) were calculated for the KF and KE for statistical analysis.

### 2.4 Statistical analysis

The statistical analysis was performed using SPSS Statistics 29.0.0.0 software (IBM, Armonk, United States). The normality of the data was tested using the Kolmogorov‒Smirnov test. In the case of non-normality, parametric tests were performed. In cases where the Kolmogorov‒Smirnov test was significant, but the values for skewness and kurtosis were below the predefined normality reference limits of <2.0 and <7.0 (West et al., 1995), the parametric tests were backed-up by bias corrected accelerated (BCa) bootstrapping with 10,000 samples. Independent-samples *t*-tests were performed to investigate sex differences in individual HAM and QUAD ACSA_max_, absolute and relative KF and KE MVTs and corresponding ratios. Sex differences in proportional ACSA_max_ (i.e., ACSA_max_ relative to the respective muscle group HAM or QUAD) were analysed using two-factor analysis of variance with the Bonferroni *post hoc* correction. The statistical significance level was set to *p* = 0.05. For statistically significant differences, the effect sizes were calculated and interpreted according to Cohen (Cohen, 1992): r = 0.2 corresponds to a small effect, r = 0.5 corresponds to a medium effect, and r = 0.8 corresponds to a large effect.

## 3 Results

### 3.1 Maximal anatomical cross-sectional areas of the hamstrings and quadriceps muscles in female and male elite competitive alpine skiers

Table 2 shows the ACSA_max_ values of the HAM and QUAD muscles in female and male elite competitive alpine skiers. Within the HAM muscles, male skiers showed a significantly greater ACSA_max_ in the BFsh (*p* = 0.006, r = 0.5), BFlh (*p* < 0.001, r = 0.6) and ST (*p* = 0.001, r = 0.5), and as an entire HAM muscle group (*p* < 0.001, r = 0.7) than female skiers. The ACSA_max_ of the SM (*p* = 0.152) did not differ significantly between the two sexes. In the QUAD muscles, male skiers had significantly greater ACSA_max_ values in the VL (*p* = 0.002, r = 0.5), RF (*p* = 0.009, r = 0.4), VM (*p* = 0.005, r = 0.5) and entire QUAD muscle groups (*p* = 0.006, r = 0.5) than female skiers did. There were no significant differences in the ACSA_max_ between the two sexes for the VI (*p* = 0.075).

**TABLE 2:** Maximal anatomical cross-sectional areas of the hamstrings and quadriceps muscles in female and male elite competitive alpine skiers (n = 33).

	Female (n = 20)	Male (n = 13)	*p*-value
HAM ACSA_max_ (cm^2^)			
BFsh	6.24 ± 1.76	8.17 ± 1.91	0.006
BFlh	12.38 ± 1.89	15.83 ± 2.41	<0.001
ST	10.92 ± 2.23	14.22 ± 3.08	0.001
SM	14.65 ± 2.14	15.88 ± 2.66	0.152
HAM	44.19 ± 5.18	54.10 ± 5.56	<0.001
QUAD ACSA_max_ (cm^2^)			
VL	29.36 ± 4.75	36.08 ± 6.66	0.002
RF	12.38 ± 2.67	15.19 ± 3.13	0.009
VM	17.98 ± 3.94	23.94 ± 6.13	0.005
VI	31.50 ± 3.61	36.24 ± 8.44	0.075
QUAD	91.22 ± 11.52	111.45 ± 20.95	0.006

The data are expressed as the mean ± standard deviation. Independent-samples t-tests were performed to investigate sex differences in HAM and QUAD ACSA_max_. The statistical significance level was set to *p* = 0.05. ACSA_max_, maximal anatomical cross-sectional area; BFsh, biceps femoris short head; BFlh, biceps femoris long head; ST, semitendinosus; SM, semimembranosus; HAM, hamstrings; VL, vastus lateralis; RF, rectus femoris; VM, vastus medialis; VI, vastus intermedius; QUAD, quadriceps.

### 3.2 Proportional maximal anatomical cross-sectional areas of the hamstrings and quadriceps muscles in female and male elite competitive alpine skiers

**FIGURE 2:**
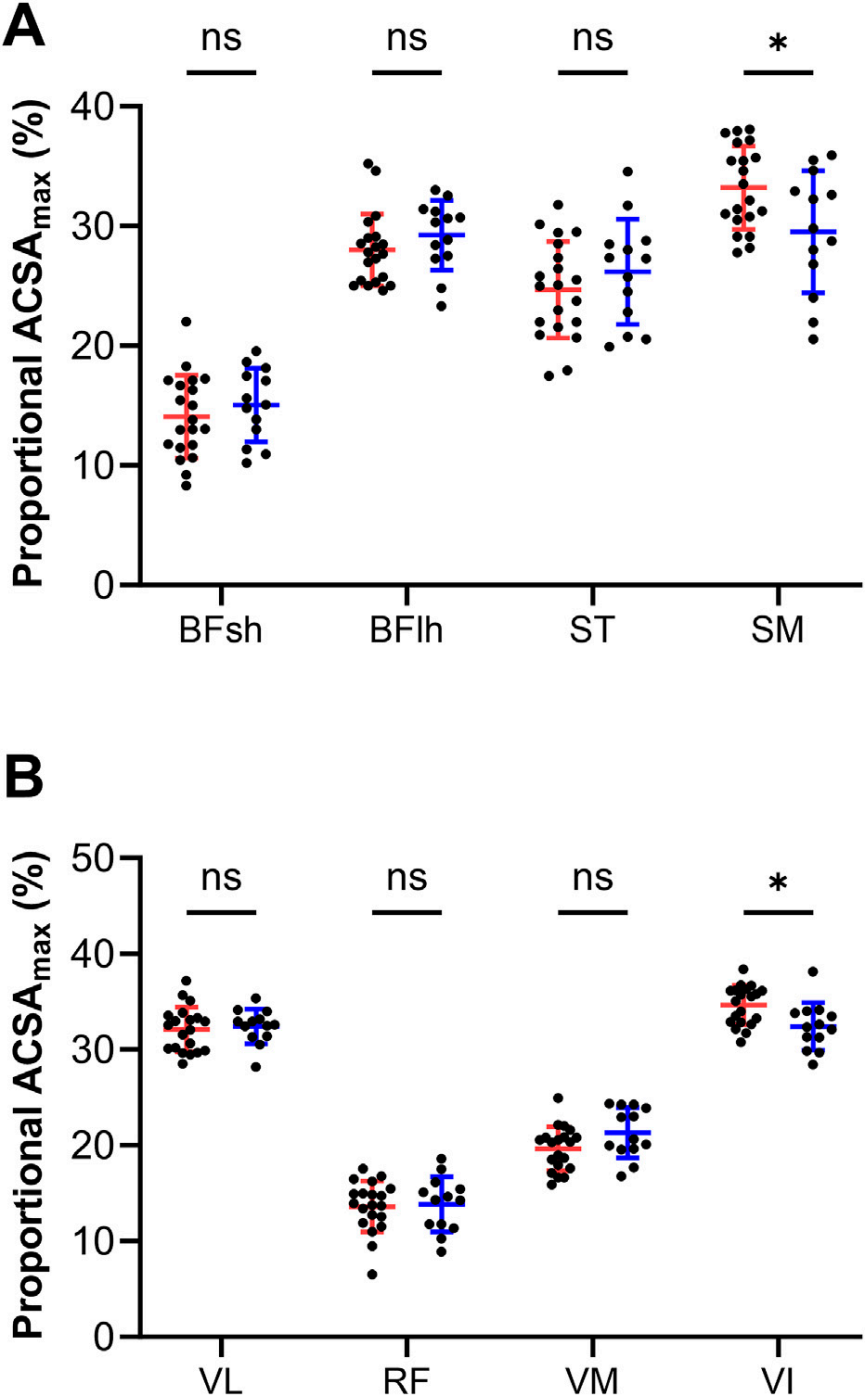
Proportional maximal anatomical cross-sectional areas of the hamstrings **(A)** and quadriceps **(B)** muscles in female (red lines) and male (blue lines) elite competitive alpine skiers. The data are expressed as the mean ± standard deviation and individual values. ACSA_max_: maximal anatomical cross-sectional area, BFsh: biceps femoris short head, BFlh: biceps femoris long head, ST: semitendinosus, SM: semimembranosus, VL: vastus lateralis, RF: rectus femoris, VM: vastus medialis, VI: vastus intermedius.

Figure 2 shows the proportional ACSA_max_ of the HAM (A) and QUAD (B) muscles in female and male elite competitive alpine skiers. Within the HAM muscles, female skiers (32.32% ± 3.94%) had a significantly greater proportional SM ACSA_max_ (*p* = 0.024, r = 0.2) than male skiers (30.92% ± 4.97%). The proportional ACSA_max_ of the BFsh (female: 14.42% ± 3.29% vs. male: 14.61% ± 3.13%) (*p* > 0.999), BFlh (female: 28.37% ± 2.94% vs. male: 29.40% ± 3.31%) (*p* > 0.999) and ST (female: 24.89% ± 3.86% vs. male: 25.08% ± 4.59%) (*p* > 0.999) did not significantly differ between the sexes. In the QUAD muscles, female skiers (33.89% ± 2.38%) had a greater proportional ACSA_max_ in the VI (*p* = 0.040, r = 0.1) than male skiers did (33.55% ± 2.79%). VL (female: 32.11% ± 2.07% vs. male: 32.17% ± 2.03%) (*p* > 0.999), RF (female: 13.66% ± 2.73% vs. male: 13.17% ± 3.01%) (*p* > 0.999) and VM (female: 20.33% ± 2.56% vs. male: 21.11% ± 2.63%) (*p* = 0.208) did not differ significantly between the two sexes in terms of the proportional ACSA_max_.

### 3.3 Knee flexion and knee extension maximal voluntary torque production in female and male elite competitive alpine skiers

**FIGURE 3:**
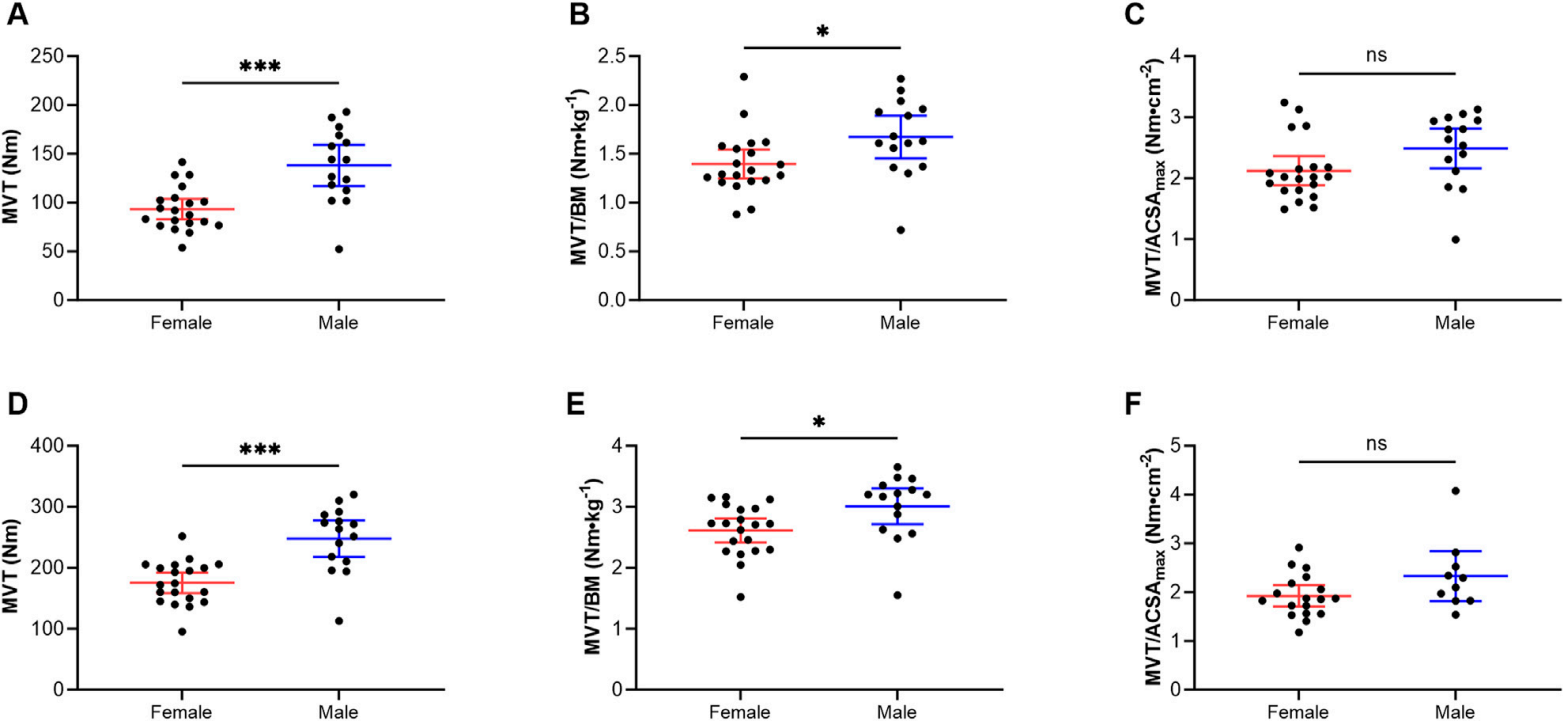
Knee flexion **(A–C)** and knee extension **(D–F)** maximal voluntary torque production in female and male elite competitive alpine skiers. The data are expressed as the mean ± standard deviation and individual values. MVT: maximal voluntary torque production, BM: body mass, ACSA_max_: maximal anatomical cross-sectional area.

Figure 3 shows the MVT, MVT/BM and MVT/ACSA_max_ for the KF (A-C) and KE (D-F) of female and male elite competitive alpine skiers. For the KF MVT, male skiers (138.0 ± 38.1 Nm) had significantly greater values than female skiers did (93.5 ± 22.2 Nm) (*p* < 0.001, r = 0.6). This was also the case for the KF MVT/BM, where male skiers (1.7 ± 0.4 Nm·kg^−1^) displayed significantly greater values than female skiers did (1.4 ± 0.4 Nm·kg^−1^) (*p* = 0.029, r = 0.4). There was no significant difference for the KF MVT/ACSA_max_ between the two sexes (female: 2.1 ± 0.5 Nm·cm^−2^ vs. male: 2.5 ± 0.6 Nm·cm^−2^) (*p* = 0.057). For the KE MVT, male skiers (248.0 ± 54.2 Nm) had also significantly greater values than female skiers did (175.0 ± 35.8 Nm) (*p* < 0.001, r = 0.6). This was also the case for the KE MVT/BM, where male skiers (3.0 ± 0.5 Nm·kg^−1^) displayed significantly greater values than female skiers did (2.6 ± 0.4 Nm·kg^−1^) (*p* = 0.019, r = 0.4). However, there was no significant difference between the two sexes (female: 1.9 ± 0.4 Nm·cm^−2^ vs. male: 2.3 ± 0.7 Nm·cm^−2^) for MVT/ACSA_max_ (*p* = 0.074).

### 3.4 Maximal anatomical cross-sectional area ratio of the hamstrings-to-quadriceps muscles and maximal voluntary torque production ratio of knee flexion-to-knee extension in female and male elite competitive alpine skiers

**FIGURE 4:**
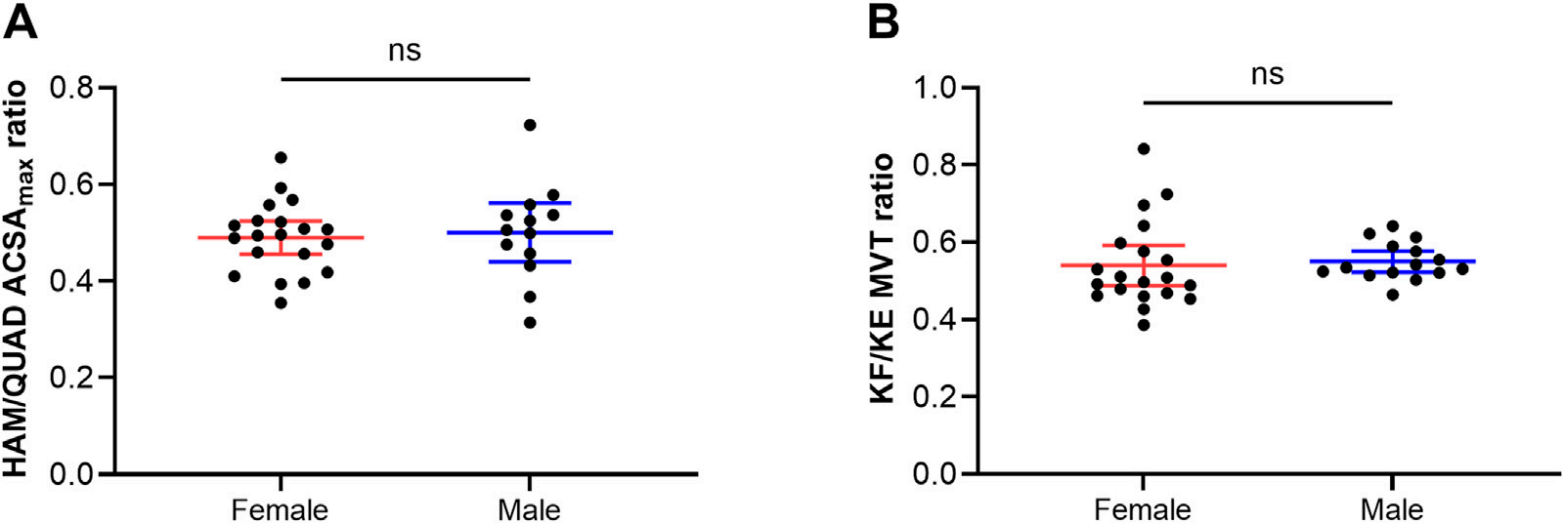
Hamstrings-to-quadriceps maximal anatomical cross-sectional area ratio **(A)** and knee flexion-to-knee extension maximal voluntary torque production ratio **(B)** in female and male elite competitive alpine skiers. The data are expressed as the mean ± standard deviation and individual values. HAM: hamstrings, QUAD: quadriceps, ACSA_max_: maximal anatomical cross-sectional area, KF: knee flexion, KE: knee extension.

Figure 4 shows the HAM/QUAD ACSA_max_ ratio (A) and the KF/KE MVT ratio (B) of female and male elite competitive alpine skiers. Neither the HAM/QUAD ACSA_max_ ratio (female: 0.5 ± 0.1 vs. male: 0.5 ± 0.1) (*p* = 0.726) nor the KF/KE MVT ratio (female: 0.5 ± 0.1 vs. male: 0.6 ± 0.1) (*p* = 0.745) differed significantly between the two sexes.

## 4 Discussion

The main findings were as follows: (1) male skiers had greater ACSA_max_ values for the BFsh, BFlh, ST, VL, RF, and VM muscles than female skiers did, but no significant differences between the sexes were observed for the SM and VI; (2) conversely, female skiers had a greater proportional ACSA_max_ for the SM and VI than male skiers did, whereas no significant differences between the sexes were observed for the BFsh, BFlh, ST, VL, RF, and VM; (3) male skiers had greater KF and KE MVT values in absolute terms and normalized to body mass than female skiers did, but the MVT/ACSA_max_ did not differ significantly between the sexes; and (4) neither the HAM/QUAD ACSA_max_ ratio nor the KF/KE MVT ratio showed significant differences between the sexes.

It is well documented in the literature that female athletes display lower average muscle mass than male athletes do (Bassett et al., 2020; Hunter et al., 2023). In the present study, this was the case for the individual muscles BFsh, BFlh, ST, VL, RF, and VM as well as in total as a muscle group (i.e., for the HAM and the QUAD), where female skiers had a lower ACSA_max_ than male skiers did. Compared with a study with moderately physically active participants (Behan et al., 2018), the female and male competitive alpine ski racers in the present study had greater ACSA_max_ values for the HAM and QUAD muscles (individually and as a group). In addition, while we found no significant differences between the sexes for SM and VI in the present study, (Behan et al., 2018) reported that male participants had greater ACSA_max_ values for each individual HAM and QUAD muscle than females did. Although, in general, lower ACSA values are expected to be found in females than in males, a high level of training seems to decrease these differences between the sexes for some muscles.

In the field of competitive alpine skiing, however, a comparison of the reference values from the current study is not possible because, to the best of our knowledge, no ACSA_max_ data are currently available for a comparable cohort. At best, only a previously published study (Franchi et al., 2020) by our research group in which the HAM ACSAs of 85 youth competitive alpine skiers were examined can be used for comparison, whereby the age and level of these skiers were significantly lower. For BFsh, BFlh, ST and SM, youth skiers (14.8 ± 0.5 years) presented ACSA_max_ values of 5.7 ± 1.4 cm^2^, 10.4 ± 2.2 cm^2^, 8.6 ± 2.0 cm^2^ and 10.1 ± 2.3 cm^2^ respectively. The elite competitive alpine skiers in the present study presented, on average, higher ACSA_max_ values for all HAM muscles. This underlines the substantial changes in the ACSA_max_ of the individual HAM muscles that competitive alpine skiers undergo during growth. The ACSA_max_ values obtained in the present study can also be used as a benchmark for youth competitive alpine skiers striving towards the elite level. With respect to QUAD, a muscle group that is considered highly developed in competitive alpine skiers, it may be reasonable to compare the ACSA_max_ values to those of experienced resistance training practitioners. Young men who underwent long-term resistance training (i.e., systematic, progressive, heavy QUAD resistance training ∼3/week for 3 years) had ACSA_max_ values of 135.0 ± 15.0 cm^2^ (Maden-Wilkinson et al., 1985). A group of young men with a similar resistance training history had an average ACSA_max_ value of 138 ± 14 cm^2^ (Balshaw et al., 2022). Although QUAD muscles are considered highly developed in elite competitive alpine skiers, the male skiers in the present study presented to some extent lower ACSA_max_ values on average.

In terms of the proportional ACSA_max_, the SM of female skiers within the HAM was greater than that of male skiers. This is noteworthy because, in addition to flexion of the knee joint and extension of the hip joint, the SM also acts as an internal rotator of the tibia (Cleather, 2018). Since internal rotation of the tibia relative to the femur is a common injury mechanism in competitive alpine skiing (Spörri et al., 2017; Jordan et al., 2017), this result could be important for preventing ACL injuries. The fact that the SM accounts for a greater proportion of the HAM in female skiers than in male skiers could theoretically mean that internal rotation of the tibia is more likely to occur when the HAM is activated during knee flexion, which could result in more ACL strain. In a study by Behan et al. (2018) with low-moderate physically active volunteers, female participants also had a greater proportional ACSA_max_ for SM than male participants did. Conversely, however, the female participants also presented a greater proportional ACSA_max_ for the BFlh, which, given its tibial external rotation function (Cleather, 2018), indicates a more balanced ratio of medial to lateral HAM in this sample. Thus, based on these observations and considerations, screening for the proportional ACSA_max_ of the medial (i.e., SM) and lateral (i.e., BFlh) HAM muscles may be helpful for tailoring exercise selection for the purpose of preventing ACL injuries. The practical implication of this finding could be that female skiers should aim to balance the ratio between medial HAM and lateral HAM by especially performing exercises that target the lateral portion of the HAM.

The KF and KE MVT values determined in the present study were lower both in absolute terms and relative to body mass than the values reported in previous studies (Neumayr et al., 2003; Jordan et al., 2015). In a study of 20 female and 28 male alpine skiers from the Austrian World Cup ski team (Neumayr et al., 2003), for example, who were tested once a year over a period of 3 years, the skiers presented higher absolute KF and KE MVT values in comparison. A study of 13 male and 8 female skiers from the Canadian alpine ski team (Jordan et al., 2015) also showed higher body mass normalised MVT values for KF and KE than the skiers from the present study. A comparison between the sexes revealed that male skiers presented both greater absolute and relative KF and KE MVTs than females did. This is in contrast to the study from Jordan et al. (2015), which found no sex differences in body mass-normalised MVTs. The MVT relative to the corresponding ACSA_max_ of the HAM or QUAD muscle group did not differ between the two sexes, indicating comparable “muscle quality” between the two sexes. This finding is consistent with the study by Castro et al. (Castro et al., 1995), which revealed no sex differences in peak torque/CSA in untrained and trained adults.

Neither the muscle ratio (i.e., HAM/QUAD ACSA_max_ ratio) nor the torque ratio (i.e., KF/KE MVT ratio) differed between the two sexes in the present study. With respect to the muscle ratio, a study by Behan et al. (2018) reported an average lower value in females than in males. However, in comparison to the present study, the authors also included the sartorius and gracilis muscles for the KF, as these muscles are also involved in KF. Given that, according to a systematic and critical review (Kellis et al., 2023), the KF/KE MVT ratio has limited value for predicting only ACL injuries, the question arises *a priori* whether this ratio is relevant in the context of ACL injury prevention. Nonetheless, one of the strengths of the present study from our point of view was that we collected both the muscle and torque ratios, and therefore we investigated whether there were sex-specific differences between the two ratios.

The present study has several limitations that should be considered. The sample size was relatively small, especially because it was divided by sex. Accordingly, some of the potential differences assessed were, despite large effect sizes, not statistically significant due to limited statistical power. Moreover, the ultrasound and dynamometer measurements were carried out on different days and not all participants completed both measurements, which can lead to a certain degree of variability. The measurements were limited to the right leg only. Care should be taken when comparing the parameter values of our study with those of other studies, as there may be some differences in the methodology used. Finally, the study design and the lack of data on ACL injuries do not allow any direct conclusions to be drawn about the role of the measured variables in injury prevention.

## 5 Conclusion

The present study provides normative values for the muscle size and strength of the HAM and QUAD muscles of elite competitive alpine skiers. These values can be used as benchmarks for youth competitive alpine skiers striving for the elite level. Based on the literature, it was expected that males would present a greater ACSA_max_ in most HAM and QUAD muscles and a greater absolute MVT in the KF and KE. An interesting finding of the present study was that female skiers had a greater proportional ACSA_max_ of the SM, as this may be relevant for ACL injury prevention given the function tibia internal rotation.

## Data Availability

The datasets presented in this article are not readily available because their access is restricted to protect the interests of the project partner Swiss-Ski and their athletes. Requests to access the datasets should be directed to joerg.spoerri@balgrist.ch.
